# Direct integrin binding to insulin-like growth factor-2 through the C-domain is required for insulin-like growth factor receptor type 1 (IGF1R) signaling

**DOI:** 10.1371/journal.pone.0184285

**Published:** 2017-09-05

**Authors:** Dora Maria Cedano Prieto, Yushen Cheng, Chih-Chieh Chang, Jessica Yu, Yoko K. Takada, Yoshikazu Takada

**Affiliations:** 1 Department of Dermatology, Biochemistry and Molecular Medicine, UC Davis School of Medicine, Research III Suite, 4645 Second Avenue, Sacramento, CA, United States of America; 2 College of Medical Science and Technology, Taipei Medical University, Taipei, Taiwan; 3 Taiwan Protein Project (TPP), Academia Sinica, Sec. 2, Nankang, Taipei, Taiwan, R.O.C; Seoul National University College of Pharmacy, REPUBLIC OF KOREA

## Abstract

We have reported that integrins crosstalk with growth factors through direct binding to growth factors (e.g., fibroblast growth factor-1, insulin-like growth factor 1 (IGF1), neuregulin-1, fractalkine) and subsequent ternary complex formation with cognate receptor [e.g., integrin/IGF1/IGF1 receptor (IGF1R)]. IGF1 and IGF2 are overexpressed in cancer and major therapeutic targets. We previously reported that IGF1 binds to integrins ανβ3 and α6β4, and the R36E/R37E mutant in the C-domain of IGF1 is defective integrin binding and signaling functions of IGF1, and acts as an antagonist of IGF1R. We studied if integrins play a role in the signaling functions of IGF2, another member of the IGF family. Here we describe that IGF2 specifically binds to integrins ανβ3 and α6β4, and induced proliferation of CHO cells (IGF1R+) that express ανβ3 or α6β4 (β3- or α6β4-CHO cells). Arg residues to Glu at positions 24, 34, 37 and/or 38 in or close to the C-domain of IGF2 play a critical role in binding to integrins and signaling functions. The R24E/R37E/R38E, R34E/R37E/R38E, and R24E/R34E/R37E/R38E mutants were defective in integrin binding and IGF2 signaling. These mutants suppressed proliferation induced by WT IGF2, suggesting that they are dominant-negative antagonists of IGF1R. These results suggest that IGF2 also requires integrin binding for signaling functions, and the IGF2 mutants that cannot bind to integrins act as antagonists of IGF1R. The present study defines the role of the C-domain in integrin binding and signaling.

## Introduction

Integrins are transmembrane receptor heterodimers formed by α and β chains. Integrins bind to extracellular matrix ligands (e.g., fibronectin, collagen, and vitronectin), cell surface ligands (e.g., intercellular adhesion molecule-1 and vascular cell adhesion molecule-1) and soluble ligands including several growth factors [1]. Several integrins, including ανβ3 and α6β4 are overexpressed in human cancers [2]. We have reported that integrins crosstalk with growth factors through direct binding to growth factors [e.g., fibroblast growth factor-1 (FGF1) [3], insulin-like growth factor 1 (IGF1) [4–6], neuregulin-1 [7], fractalkine [8]] and subsequent ternary complex formation with cognate receptor [e.g., integrin/IGF1/insulin-like growth factor type 1 receptor (IGF1R)] [9,10]. We describe this as the ternary complex model of growth factor signaling. The importance of integrins in growth factor signaling was underscored by the findings that the growth factor mutants that cannot bind to integrins are defective in signaling while they still bind to cognate receptors and are dominant-negative antagonists. In the case of IGF1, an IGF1 mutant in which the Arg residues at positions 36 and 37 to Glu mutant, R36E/R37E was defective in inducing IGF1R signaling and suppressed cell proliferation induced by WT IGF1 in vitro and tumor growth in vivo (dominant-negative antagonistic action)[11]. The dominant-negative growth factor mutants we developed have potential as therapeutic agents and are useful tools for studying the role of integrins in growth factor signaling. It is still unclear if integrins play a role in signaling functions of other members of the same growth factor family. We recently reported that the integrin-binding defective mutants of FGF2 (basic FGF) are defective in signaling functions and potently suppressed angiogenesis induced by WT FGF2, suggesting that integrins may be common co-receptors for other members of the FGF family as well [12]. These findings urged us to study if IGF2, another member of the IGF/insulin family, requires integrin binding for signaling functions.

IGF1 and IGF2 are homologues and share 67% sequence identity [13], and are involved in growth and development of many tissues in the human body and in tumor growth [14]. IGF1 and IGF2 induce cell proliferation through IGF1R [14]. IGF2 binding to the extracellular subunit of IGF1R induces phosphorylation of the tyrosine kinase of the intracellular β subunit of the receptor, resulting in the activation of MAPK pathway and PI3K/AKT pathway [14,15].

We studied the role of integrins in IGF2 signaling through IGF1R. We show that IGF2 binds to integrins ανβ3 and α6β4, and induces intracellular signaling in an integrin-dependent manner in CHO cells (IGF1R+) that overexpress these integrins. We located the integrin-binding site of IGF2 in the C-domain that connects B and A domains, suggesting that the C-domain plays a critical role in IGF signaling. The IGF2 mutants defective in integrin binding were defective in signaling and acted as antagonists of IGF1R, indicating that the IGF2 signaling also fits well with the ternary complex model.

## Results

### IGF2 directly binds to integrin ανβ3

**Fig 1:**
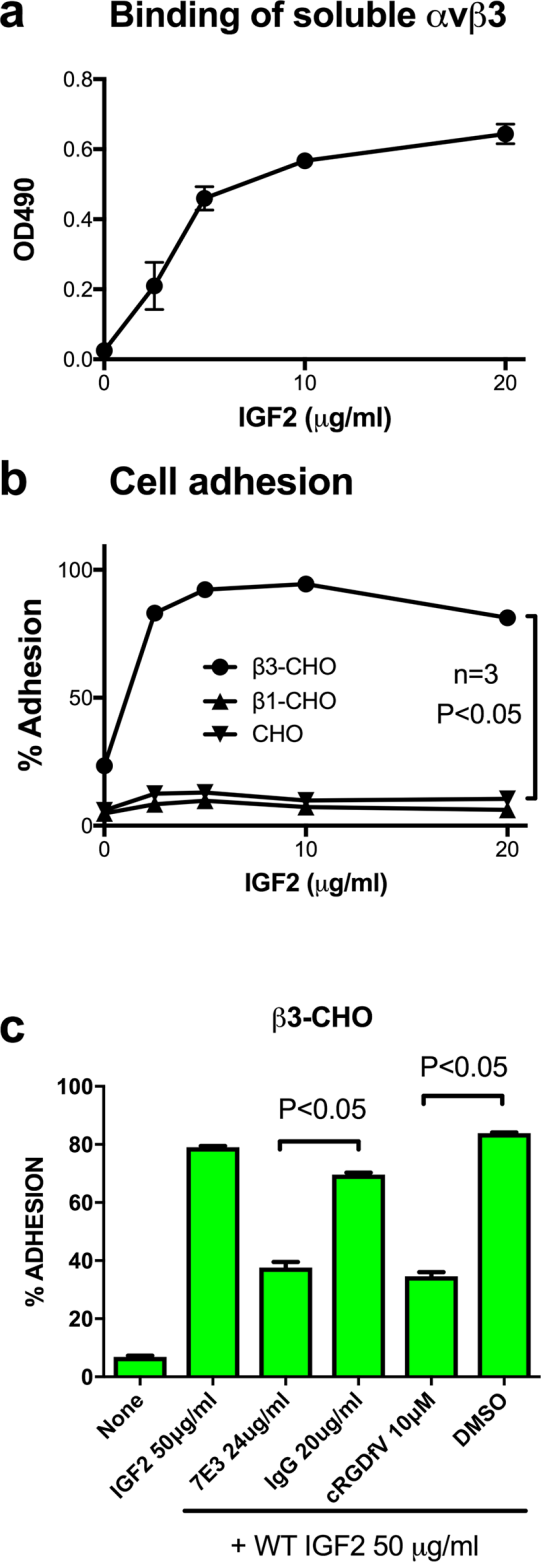
Specific binding of IGF2 to ανβ3. *a*, Binding of soluble αvβ3 to IGF2 in ELISA-type binding assays. Wells of 96 well microtiter plates were coated with increasing concentrations of IGF2. Soluble recombinant αvβ3 (5 μg/ml) was incubated with immobilized IGF2 in Tyrode-HEPES buffer containing 1 mM Mg^2+^. The αvβ3 bound to IGF2 was measured using anti-integrin β3 mAb. The data are shown as means +/- SEM of triplicate experiments. *b*, CHO cells expressing recombinant αvβ3 (β3-CHO cells) bound to IGF2 while CHO cells and CHO cells expressing αvβ1 (β1-CHO) did not. Wells 96 well microtiter plates were coated with IGF2 at increasing concentrations. The wells were incubated with β3-CHO, β1-CHO and CHO cells in serum free DMEM buffer (10^5^ cells/well). The bound cells were measured. The data are shown as means +/- SEM of triplicate experiments. *c*, Antibody against αvβ3 (7E3) and cyclic RGDfV blocked the adhesion of β3-CHO cells to IGF2. Wells 96 well microtiter plates were coated with IGF2 at 50 μg/ml. β3-CHO cells (10^5^ cells/well) were incubated with the immobilized IGF2 plus 7E3 or cyclic RGDfV in Tyrode-HEPES buffer containing 1 mM Mg^2+^, the bound cells were measured. The data are shown as means +/- SEM of triplicate experiments.

We studied if soluble integrin αvβ3 binds to immobilized IGF2 in an ELISA-type binding assay. We found that soluble αvβ3 bound to IGF2 in a dose dependent manner, but did not bind to wells coated only with BSA (Fig 1A). We studied if αvβ3 on the cell surface binds to immobilized IGF2 in adhesion assays using CHO cells that express recombinant hamster αv/human β3 hybrid (β3-CHO cells). β3-CHO cells adhered to IGF2 in a dose dependent manner, but control CHO cells that express human integrin β1 (β1-CHO) or parent CHO cells did not (Fig 1B). Monoclonal antibody (mAb) 7E3 (specific to human β3) and cyclic RGDfV (a specific inhibitor of αvβ3) reduced the ability of β3-CHO cells to bind to IGF2 in adhesion assays, but control mouse IgG or DMSO did not (Fig 1C). These results suggest that WT IGF2 specifically binds to αvβ3.

### IGF2 enhances intracellular signaling and cell proliferation in a αvβ3-dependent manner

**Fig 2:**
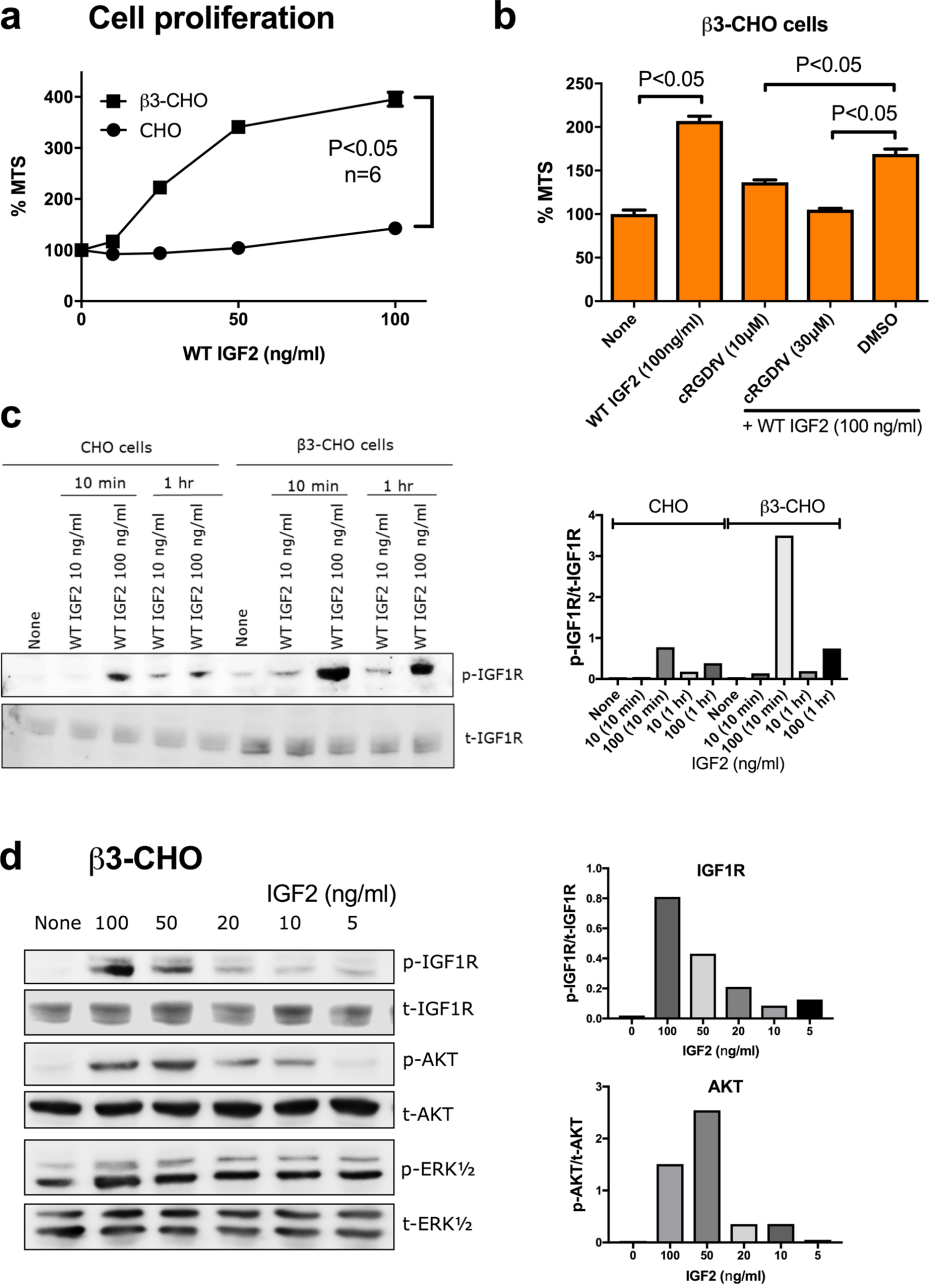
Integrin binding is required for IGF2-induced cell proliferation. *a*, Cell proliferation was enhanced by IGF2 in β3-CHO cells better than CHO cells. Cells (2 x 10^4^ cells/well) were incubated for 48 hrs in polyHEMA-coated plates. Cell proliferation was measured by MTS assays. The data are shown as means +/- SEM of triplicate experiments. *b*, IGF2-induced cell proliferation was reduced by cyclic RGDfV. Cells (2 x 10^4^ cells/well) were incubated with IGF2 (100 ng/ml) in combination with cyclic RGDfV for 48 hrs in polyHEMA-coated plates. Cell proliferation was measured by MTS assays. The data are shown as means +/- SEM of triplicate experiments. *c*. IGF2 induces phosphorylation of IGF1R in a dose and time-dependent manner. Cells were serum-starved in serum free DMEM for 4 hrs, and treated with WT IGF2 (10 or 100 ng/ml) for 10 min or 1 hr in a polyHEMA coated plates. Cell lysates were analyzed by western blotting. Density of the bands were quantified using ImageJ software and p-IGF1R/t-IGF1R was calculated. *d*, IGF2 induces signals in β3-CHO cells. Cells were serum-starved in serum free DMEM for 4 hrs, and treated with WT IGF2 for 10 minutes in a polyHEMA coated plates. Cell lysates were analyzed by western blotting. Density of the bands were quantified using ImageJ software and p-IGF1R/t-IGF1R or p-AKT/t-AKT was calculated.

We studied if IGF2-induced intracellular signaling requires αvβ3 in β3-CHO cells. All the experiments were performed in anchorage-independent conditions (using polyHEMA coated plates) to reduce signals generated by cell-extracellular matrix interaction. IGF2 induced proliferation of β3-CHO cells in a dose dependent manner to a much higher extent than in CHO cells (Fig 2A). Cell proliferation induced by WT IGF2 in β3-CHO cells was reduced by cyclic RGDfV in MTS assays (Fig 2B). These findings suggest that IGF2-induced cell proliferation is dependent on αvβ3. IGF2 induced IGF1R phosphorylation in β3-CHO cells at a higher level than in CHO cells in a time- and dose-dependent manner (Fig 2C and 2D). IGF2 induced AKT and ERK activation in β3-CHO cells in a dose-dependent manner (Fig 2D). These results suggest that αvβ3 is required for IGF2-induced signaling through IGF1R.

### Development of IGF2 mutants that are defective in binding to integrin

**Fig 3:**
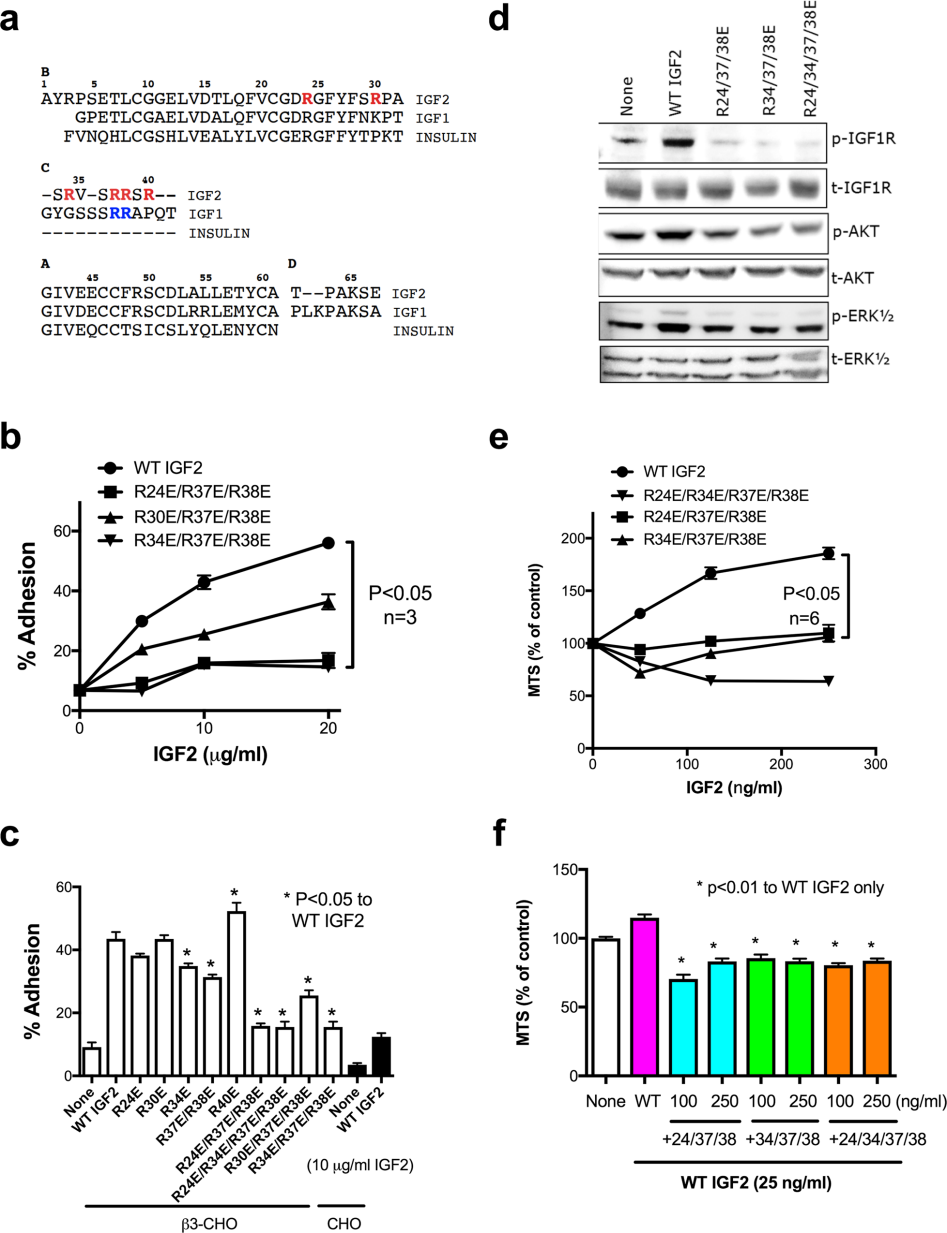
The C-domain is critically involved in integrin binding and the integrin-binding defective IGF2 mutants are defective in signaling functions and antagonistic (dominant-negative). a. Alignment of IGF1, IGF2 and insulin. Previous studies showed that the critical amino acid residues for integrin binding in IGF1 are present in the C-domain (Arg36 and Arg37, in blue) (5). Thus several Arg residues at positions 24, 30, 34, 37, 38 and 40 (in red) of IGF2 in and around the C-domain were selected for mutagenesis. b and c. Mutations in the predicted integrin-binding interface of IGF2 suppress integrin binding of IGF2. Wells of 96-well microtiter plate were coated with WT and mutant IGF2. Then β3-CHO cells and CHO cells were incubated in Tyrode-HEPES buffer containing 1 mM Mg^2+^. The bound cells were measured. The data are shown as means +/- SEM of triplicate experiments. *d*, R24E/R37E/R38E, R34E/R37E/R38E, and R24E/R34E/R37E/R38E are defective in inducing phosphorylation of the IGF1R, AKT and ERK1/2 in β3-CHO cells. Cells were treated with WT IGF2, R24E/R37E/R38E, R34E/R37E/R38E, or R24E/R34E/R37E/R38E at 100 ng/ml for 10 min. Cell lysates were analyzed by western blotting. *e*, R24E/R37E/R38E, R34E/R37E/R38E, and R24E/R34E/R37E/R38E are defective in inducing proliferation of β3-CHO in MTS assays. β3-CHO cells were incubated with WT IGF2, R24E/R37E/R38E, R34E/R37E/R38E, or R24E/R34E/R37E/R38E at increasing concentrations for 48 hrs in 96-well plate coated with polyHEMA. The data are shown as means +/- SEM (n = 6). *f*, R24E/R37E/R38E, R34E/R37E/R38E, and R24E/R34E/R37E/R38E inhibited cell proliferation induced by WT IGF2 (25 ng/ml) in MTS assays. The data are shown as means +/- SEM (n = 6).

Our results so far suggest that αvβ3 binds to IGF2 and is involved in IGF1R signaling. To further study the role of ανβ3 in IGF2 signaling, we generated IGF2 mutants that are defective in integrin binding. We previously reported that Arg36 and Arg37 in the C-domain of IGF1 are critical for integrin-binding [5]. Based on the homology between IGF2 and IGF1 (Fig 3A), we substituted Arg residues at positions 24, 30, 34, 37, 38, and 40 in and around the C-domain of IGF2 were mutated to Glutamic acid. The IGF2 mutants were tested for the ability to bind to ανβ3 in adhesion assays using β3-CHO cells. Although single point mutations did not effectively suppress integrin binding, the combined R24E/R37E/R38E, R34E/R37E/R38E, and R24E/R34E/R37E/R38E mutations effectively suppressed the binding of IGF2 to β3-CHO cells (Fig 3B and 3C). The data suggest that Arg residues at positions 24, 34, 37, and 38 in or close to the C-domain of IGF2 are critical for binding to αvβ3.

### The integrin-binding defective IGF2 mutants are defective in signaling functions and are an antagonist of IGF1R

We studied the signaling functions of the R24E/R37E/R38E, R34E/R37E/R38E, and R24E/R34E/R37E/R38E mutants using β3-CHO cells in polyHEMA-coated plates. R24E/R37E/R38E, R34E/R37E/R38E, and R24E/R34E/R37E/R38E were defective in inducing activation of IGF1R, AKT, and ERK1/2 (Fig 3D) and cell proliferation by MTS assays (Fig 3E). We studied if the integrin-binding defective IGF2 mutants act as antagonists of IGF1R. We used minimum amount of WT IGF2 (25 ng/ml) that induces detectable cell proliferation. Notably, excess IGF2 mutants suppressed cell proliferation induced by WT IGF2 (Fig 3F), suggesting that R24E/R37E/R38E, R34E/R37E/R38E, and R24E/R34E/R37E/R38E are dominant-negative antagonists of IGF1R.

### IGF2 binds to integrin α6β4 and induces proliferation of α6β4-CHO cells

**Fig 4:**
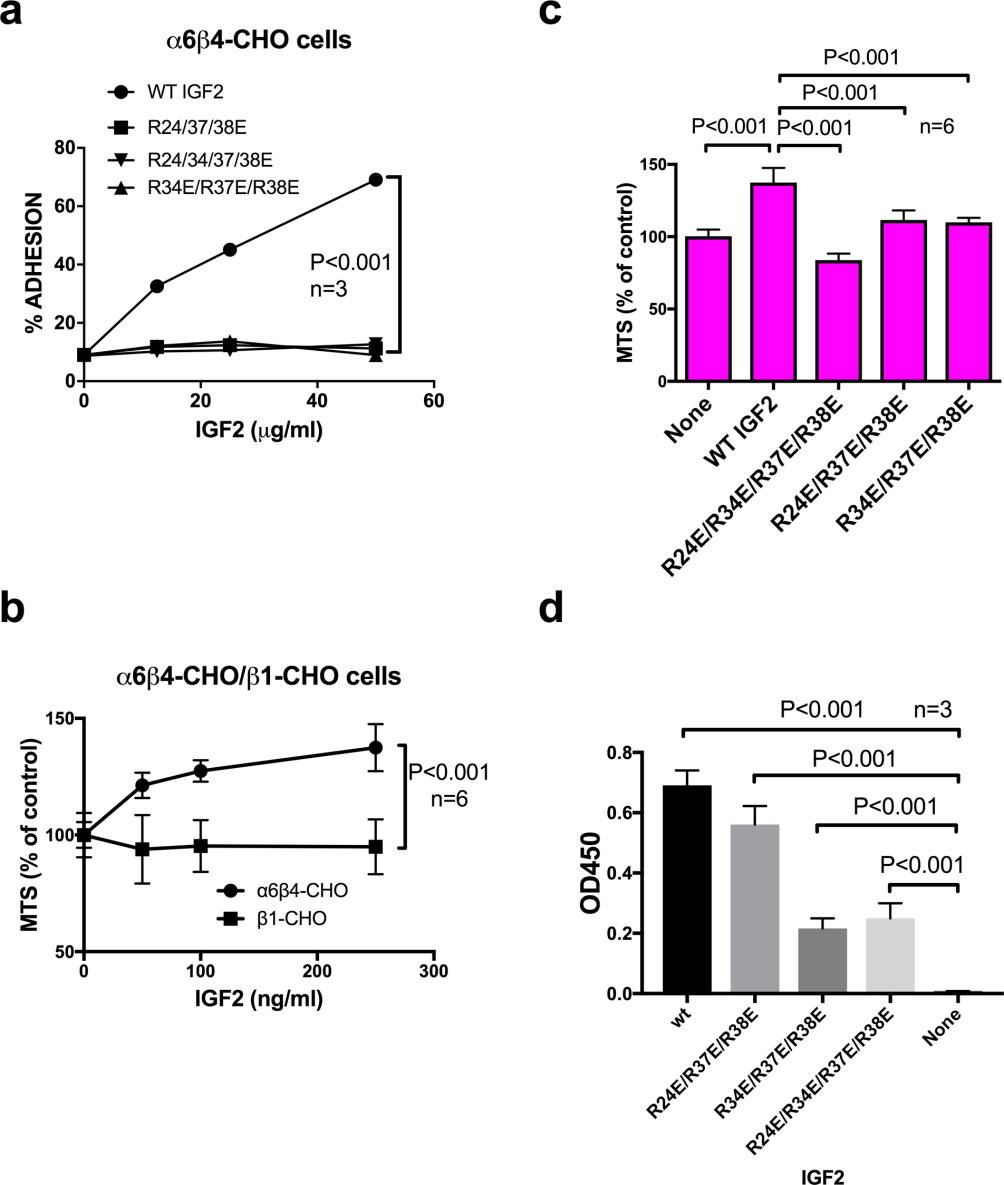
IGF2 binds to α6β4 and induces proliferation of α6β4-CHO cells while integrin-binding defective IGF2 mutants are defective in these functions. *a*, α6β4-CHO cells bind to WT IGF2 in adhesion assay. Wells of 96-well microtiter plates were coated with WT IGF2. α6β4-CHO cells were incubated in Tyrode-HEPES buffer containing 1 mM Mg^2+^, and bound cells were measured. The data are shown as means +/- SEM of triplicate experiments. *b*, WT IGF2 enhanced proliferation of α6β4-cells but not β1-CHO cells. α6β4-CHO and β1-CHO cells were incubated for 48 hrs with increasing concentrations of WT IGF2 in polyHEMA-coated plates. The data are shown as means +/- SEM (n = 6). *c*, Integrin-binding defective IGF2 mutants were defective in inducing proliferation of α6β4-CHO cells in MTS assays while WT IGF2 induced it. α6β4-CHO cells (2 x 10^4^ cells/well) were incubated for 48 hrs with WT or mutant IGF2 (250 ng/ml) in polyHEMA-coated plates. The data are shown as means +/- SEM (n = 6). *d*, Binding of IGF2 mutants to the immobilized IGF1R ectodomain. Wells of 96-well microtiter plate were coated with recombinant human soluble IGF1R at 1 μg/ml in PBS for 1 h at 37°C, and the remaining protein-binding sites were block by incubating with 1 mg/ml BSA for 1 h at room temperature. WT and mutant IGF2 (2.5 μg/50 μl in PBS) were added to the wells and incubated in PBS/0.05% Tween 20 at room temperature for 1 hr. After washing with PBS/0.05% Tween 20, wells were incubated with anti-5His antibody conjugated with HRP, then peroxidase substrate. The data are shown as means +/- SEM (n = 3)

Our previous studies suggested that α6β4, another integrin that is overexpressed in cancer cells, is critically involved in IGF1/IGF1R signaling through direct binding to IGF1 [6]. We studied if α6β4 is involved in IGF2 signaling. We found that WT IGF2 bound to α6β4-CHO cells in adhesion assays, but R24E/R37E/R38E, R34E/R37E/R38E, and R24E/R34E/R37E/R38E did not (Fig 4A), suggesting that α6β4 also binds to IGF2 and recognizes the C-domain of IGF2. WT IGF2 induced proliferation of α6β4-CHO cells, but not β1-CHO cells (Fig 4B). R24E/R37E/R38E, R34E/R37E/R38E, and R24E/R34E/R37E/R38E were defective in inducing proliferation of α6β4-CHO cells in MTS assays (Fig 4C). These results suggest that the binding of α6β4 to WT IGF2 through the C-domain plays a critical role in IGF2 signaling through IGF1R.

We studied if the IGF2 mutants bind to the immobilized soluble IGF1R ectodomain in ELISA-type assays. We detected the binding of WT and three IGF2 mutants to IGF1R. R24E/R37E/R38E bound to IGF1R to the similar extent to that of WT IGF2, but the binding of R34E/R37E/R38E and R24E/R34E/R37E/R38E was weaker than that of WT IGF2 (Fig 4D). We showed that the three integrin-binding defective IGF2 mutants when added in excess suppressed signaling induced by WT IGF2 (dominant-negative effect) (Fig 3B). The dominant-negative effect requires that the IGF2 mutants bind to IGF1R. It is likely that the reduced IGF1R binding of R34E/R37E/R38E and R24E/R34E/R37E/R38E is still enough to induce the dominant-negative effect.

## Discussion

In the present study, we establish that IGF2 specifically binds to ανβ3 and induced proliferation of β3-CHO cells and cyclic RGDfV suppressed the cell proliferation induced by IGF2. This suggests that the binding of IGF2 to integrin ανβ3 plays a role in IGF2/IGF1R signaling, as in the case of IGF1 [5], and IGF2 signaling fits well with the ternary complex model of growth factor signaling. The present findings also suggest that similar strategy can be used in general to identify dominant-negative antagonists of growth factors if they fit with the ternary complex model.

The biological role of the C-domains of IGF1 and IGF2 has not been established. In our previous studies, IGF1 binds to integrins ανβ3, amino acid residues of IGF1 critical for integrin binding were localized in the C-domain (Arg residues at positions 36 and 37) [5], which are conserved between IGF2 and IGF1. In the present study, we establish that Arg residues at positions 24, 34, 37, and 38 in and close to the C-domain play a critical role in integrin binding, suggesting that the C-domain of IGF2 is involved in integrin binding as in IGF1. Consistently, the C-domains of IGF1 and 2 are evolutionally as conserved as the B- and A-chains, in contrast to the less conserved C-chain of proinsulin, consistent with the idea that the C-domains of IGF1 and IGF2 play an important role in signaling. Our previous studies show that IGF1 induces integrin/IGF1/IGF1R ternary complex formation on the cell surface [5,6]. Therefore, it is highly likely that the C-domain is exposed to the surface when IGF1 and IGF2 bind to IGF1R and becomes accessible to integrins. Interestingly, Drosophila insulin-like peptide-6 (DILP6) contains a short C-domain that is well conserved and contains conserved Arg/Lys residues [16]. This suggests the possibility that DILP6 C-domain may interact with integrins and DILP6 may have similar properties to those of human IGFs.

We used the integrin-binding defective IGF2 mutants (R24E/R37E/R38E, R34E/R37E/R38E, and R24E/R34E/R37E/R38E) to define the role of integrins in IGF2 signaling. The IGF2 mutants were defective in activating IGF1R and in inducing intracellular signaling and cell proliferation in β3-CHO cells. These findings suggest that IGF2 requires integrin binding to induce signaling through IGF1R, as in the case of IGF1 [5]. We previously reported that an IGF1 mutant defective in integrin binding (R36E/R37E) acted as a dominant negative antagonist of IGF1R [11]. Excess IGF2 mutants reduced cell proliferation induced by WT IGF2 in β3-CHO cells, suggesting that the integrin-binding defective IGF2 mutants are dominant-negative antagonists of IGF1R. Taken together, our studies indicate that integrin ανβ3 is involved in IGF2 signaling through IGF1R. We also found that integrin α6β4 bound to WT IGF2 but not to R24E/R37E/R38E, R34E/R37E/R38E, and R24E/R34E/R37E/R38E, and that WT IGF2 induced proliferation of α6β4-CHO cells but not control β1-CHO cells, and that WT IGF2 induced proliferation of α6β4-CHO cells but not the IGF2 mutants. The results suggest that integrin α6β4 is also involved in IGF2 signaling through IGF1R activation. These findings suggest that integrins (e.g., ανβ3 and α6β4) are common co-receptors for IGF1 and IGF2 signaling and that the C-domain is critically involved in integrin binding and that this property is conserved between IGF1 and IGF2. Insulin, which is homologous to IGF1 and IGF2, has no C-domain and thus it would be interesting to study if integrins play a role in insulin signaling in future studies.

## Materials and methods

### Materials

Chinese hamster ovary (CHO), and MBA-MB-231 human breast cancer cells were obtained from American Type Culture Collection (ATCC). CHO cells expressing human integrin β1 (β1-CHO) or human integrin β3 (β3-CHO) were described [17]. CHO cells expressing human integrin α6β4 (α6β4-CHO) were described [7]. Anti-phospho-IGF1Rβ (Tyr-1135 and Tyr1136), anti-IGF1Rβ, anti-phospho-AKT (Ser-473), anti-AKT, anti-phospho-ERK (Thr-202 and Tyr-204), anti-ERK, actin and anti-integrin β3 antibodies were purchased from Cell Signaling Technology, Inc. (Danvers, MA). The Goat anti-rabbit IgG (H+L)-HRP conjugate (secondary antibody) was purchased from BioRad. HRP-conjugated anti-His tag (C-terminal) antibody was purchased from Qiagen (Valencia, CA). Cyclic RGDfV [18] was purchased from Enzo Life Sciences (Plymouth Meeting, PA). Poly (2-hydroxyethyl methacrylate) (Poly-HEMA) was purchased from Santa Cruz Biotechnology. The antibody 7E3 (anti-human integrin β3) was obtained from ATCC. Purified mouse IgG was purchased from Sigma. Recombinant soluble integrin ανβ3 was described [19]. PEI is linear polyethylenimine (MW 25,000) from Polyscience, Inc.

### Synthesis of IGF2

A cDNA fragment encoding WT IGF2 was amplified by PCR with synthetic oligonucleotides 5-gtggtgctcgagctcggacttggcgggggtagc-3 and 5-ccgacgcatccatggctgcttaccgccccagtgag-3 using human placenta cDNA library as a template. The PCR fragment was digested with NcoI and XhoI and subcloned into the NcoI/XhoI site of PET28a. The expression construct encodes IGF2 (residues 322–523) with a His6 tag at the C terminus, AYRPSETLCGGELLVDTLQFVCGDRGFYFSRPASSRVSRRSRGIVEECCFRSCDLLALLETYCATPAKSE. Protein was synthesized as insoluble protein by isopropyl β-d-thiogalactoside (IPTG) induction in Escherichia coli BL21. The C-terminal His tag of the protein was used to purify the protein with nickel-nitrilotriacetic acid affinity chromatography under denaturing conditions (in 8 M urea). The nickel-nitrilotriacetic acid resin was washed with 1% Triton X-114 before eluting the bound protein to eliminate endotoxin. Purified proteins were refolded in vitro following the protocols (“Isolation of proteins from inclusion bodies” available from the Björkman laboratory). In brief, the purified proteins were eluted in 8 M urea. After elution, the proteins were diluted into refolding buffer (100 mM Tris-HCl, pH 8.0, 400 mM L-Arg, 2 mM EDTA, 0.5 mM oxidized glutathione, 5 mM reduced glutathione and protease inhibitors) on ice. The dilution was kept for 16 hrs at 4°C with a slow stirring movement. Then the proteins were concentrated by ultrafiltration. Around 2 milligrams of purified proteins were obtained from 1 liter of bacterial culture. WT IGF2 protein concentration was determined by measuring A_280_.

### Signaling assays

The anchorage-independent conditions were used to perform all the signaling assays. Wells of microtiter plates were coated with 1.2 mg/cm^2^ Poly(2-hydroxyethyl methacrylate) (poly-HEMA) following the protocol as described [20]. Cells were cultured in normal conditions using regular tissue culture plate until 80–90% confluence in DMEM 10% FBS, 1% antibiotics and 1% non-essential amino acids at 37°C in 5% CO_2_ atmosphere. The cells were collected and plated in poly-HEMA plate for starvation for 4 hrs in DMEM without FBS. Serum-starved cells were treated with WT IGF2 or IGF2 mutants (R24E/R37E/R38E, R34E/R37E/R38E, and R24E/R34E/R37E/R38E). After treatment, cells were collected by spinning the samples at 3.5 rpm and then solubilized using lysis buffer (20 mM HEPES pH 7.4, 10% glycerol, 100 mM NaCl, 1 mM MgCl_2_, 1% Nonidet P-40, 20 mM NaF, 1 mM PMSF, protease inhibitor mixture (Sigma-Aldrich) and 1 mM Na_3_VO_4_). BSA assay (Thermo Scientific) was performed to determine the protein concentration to each sample. The cell lysates were analyzed by western blotting using different antibodies. The HRP-conjugated anti-IgG antibody, Supersignal West Pico and Femto (Thermo Scientific) were used to detect IgG. Fuji LAS 4000 mini luminescent image analyzer and Multi Gauge V3.0 software (Fujifilm, Tokyo, Japan) were used for analysis of the images.

### MTS assays

Cells (2x10^4^ cells/well) were serum-starved overnight in serum free media (DMEM) and incubated with proteins (WT or IGF2 mutant) for 24 hrs in 96 wells plate coated with 1.2 mg/cm^2^ of poly-HEMA following the protocol as described [20]. Cell proliferation was measured by MTS assay using Aqueous cell proliferation assay kit (Promega, Madison, WI).

### Binding to IGF1R in ELISA-type assays

Wells of 96-well microtiter plate were coated with recombinant human soluble IGF1R (391-GR, No His-tag, R&D System) at 1 μg/ml in PBS for 1 h at 37°C, and the remaining protein-binding sites were block by incubating with 1 mg/ml BSA for 1 h at room temperature. WT and mutant IGF2 (2.5 μg/50 μl in PBS) were added to the wells and incubated in PBS/0.05% Tween 20 at room temperature for 1 hr. After washing with PBS/0.05% Tween 20, wells were incubated with anti-5His antibody conjugated with HRP (Qiagen), then peroxidase substrate.

### Other methods

Cell adhesion assays [11] and binding assays [8] were performed as described. Statistical significance was tested in Prism 7 (GraphPad Software) using analysis of variance (ANOVA) and Tukey’s multiple-comparison test to control the global type I error.
